# Do Tropical Cyclones Shape Shorebird Habitat Patterns? Biogeoclimatology of Snowy Plovers in Florida

**DOI:** 10.1371/journal.pone.0015683

**Published:** 2011-01-12

**Authors:** Matteo Convertino, James B. Elsner, Rafael Muñoz-Carpena, Gregory A. Kiker, Christopher J. Martinez, Richard A. Fischer, Igor Linkov

**Affiliations:** 1 Department of Agricultural and Biological Engineering-IFAS, University of Florida, Gainesville, Florida, United States of America; 2 Risk and Decision Science Group, Engineer Research and Development Center (ERDC), United States Army Corps of Engineers (USACE), Concord, Massachusetts, United States of America; 3 Department of Geography, Florida State University, Tallahassee, Florida, United States of America; 4 Environmental Laboratory, Engineer Research and Development Center (ERDC), United States Army Corps of Engineers (USACE), Vicksburg, Mississippi, United States of America; 5 Department of Engineering and Public Policy, Carnegie Mellon University, Pittsburgh, Pennsylvania, United States of America; University of Glamorgan, United Kingdom

## Abstract

**Background:**

The Gulf coastal ecosystems in Florida are foci of the highest species richness of imperiled shoreline dependent birds in the USA. However environmental processes that affect their macroecological patterns, like occupancy and abundance, are not well unraveled. In Florida the Snowy Plover (*Charadrius alexandrinus nivosus*) is resident along northern and western white sandy estuarine/ocean beaches and is considered a state-threatened species.

**Methodology/Principal Findings:**

Here we show that favorable nesting areas along the Florida Gulf coastline are located in regions impacted relatively more frequently by tropical cyclones. The odds of Snowy Plover nesting in these areas during the spring following a tropical cyclone impact are seven times higher compared to the odds during the spring following a season without a cyclone. The only intensity of a tropical cyclone does not appear to be a significant factor affecting breeding populations.

**Conclusions/Significance:**

Nevertheless a future climate scenario featuring fewer, but more extreme cyclones could result in a decrease in the breeding Snowy Plover population and its breeding range. This is because the spatio-temporal frequency of cyclone events was found to significantly affect nest abundance. Due to the similar geographic range and habitat suitability, and no decrease in nest abundance of other shorebirds in Florida after the cyclone season, our results suggest a common bioclimatic feedback between shorebird abundance and tropical cyclones in breeding areas which are affected by cyclones.

## Introduction

The Florida Gulf coast is an invaluable resource of great biotic diversity, including shoreline-dependent birds [1], [2]. Its vast extent spans diverse coastal habitat types persisting under varying environmental conditions dictated by climate, hydrology, and land-use. However these diverse habitats reveal similarities in their apparent large-scale geological composition (such as mineralogy of beach stretches), and in common meteorological controls that drive the occupancy distribution of avifauna in space and time. Migratory birds like the Red Knot (*Calidris canutus*) utilize Florida shores mostly as stopover areas en route to their central and south America wintering grounds, or when returning north for the summer breeding season. Other shoreline-dependent birds, like the Piping Plover (*Charadrius melodus*) use these same beaches only during the winter. By contrast, the Snowy Plover (*Charadrius a. nivosus*) [3] is one of the few resident shorebirds and it has evolved traits adapted to the local conditions. The white plumage strongly suggests adaptation of SP to the white fine sandy beaches [4]–[6]. Thus the conservation of the coastal habitat is critical for this, and other, shorebird species survival.

The study of avifauna and climatological variables is not new in scientific literature. [7] studied the avian response to hurricanes in some selected forested areas along the east coast of the USA and found a decrease in community similarity and abundance but invariance of species richness for the post-cyclones patterns. [8], [9], and [10] found a decrease in the abundance of some species or a complete disappearance of other species on island ecosystems after cyclone events for selected hurricanes (hurricane Hugo in U.S. Virgin Islands, hurricane Gilbert in Jamaica, and hurricane Georges in Puerto Rico). However all the mentioned studies are about forest-dependent birds whose habitat can be significantly damaged by cyclones via high tree-mortality [11]. [12] investigated the habitat flooding risk due to projected sea-level rise of six coastal birds in north-west Europe. Using historical data, these authors found the probability of flooding increased in 50 years because of sea-level rise. [13] and [14] emphasized the importance of understanding how the ecological niche of species evolves under the influence of climatic controls over time. The frequency of climatic extremes is often more or equally important than their mean value in shaping the range of species [14]. The challenge is offered by the macroecological approach that considers the relationships between climatic and species patterns on large-scales.

Snowy Plover (SP) is a small, Florida-threatened shorebird species [15] whose disjunct population distribution is not understood. In the USA they breed in areas along the coasts of California, Oregon, Texas, Louisiana, and in some inland lacustrine/riverine areas of California, Oregon, Utah, New Mexico, Texas, and Kansas. SP also breeds and winters along the barrier islands and estuarine beaches of the Florida Gulf coast [16], [17], where they are among the most imperiled shorebirds of the region [18]–[23]. However Florida SP are genetically indistinct from the other US mainland SP [24]. Florida SP nests on beaches characterized by medium to fine white alkaline sand and silt substrate [25] common in the high-energy coastlines along the Gulf coast [16], [17]. Nesting occurs between April and late June, while the tropical cyclone season begins in June and ends in November. A cyclone is classified as a tropical depression, tropical storm or hurricane depending on its lifetime-maximum wind speed when it affected a particular coastal region. Since nesting and tropical cyclone seasons typically do not overlap, juveniles fledge before the storms arrive and subsequently are able to seek inland protection with the adults during the storms [26], [27]. However, tropical cyclones modify the beach profile by redistributing sand from the dunes to new forefront areas, and creating ephemeral pools and large overwash fans that significantly increase both breeding and brood-rearing habitats [28]–[31]. The open sparsely vegetated habitats created by cyclones are the preferred habitat by nesting SP. Dune sands show equivalent chemical and granulometric properties of the eroded sand in the pre-cyclone beach.

Processes leading to species occupancy patterns are often difficult to untangle [32]. Yet understanding them is necessary before projecting future mortality rates affected by climate change scenarios [14]. Here, we empirically assess the relationship between tropical cyclone occurrence and SP nesting areas along the Florida Gulf coast to better understand the environmental processes affecting viability of this imperiled species. We begin by showing that nesting habitats for SP occur in areas along the coast that are more frequented by tropical cyclones. We then show that the probability of a region becoming a spring nesting ground is higher if the region was affected by a tropical cyclone 7 to 10 months earlier.

## Results

**Figure 1:**
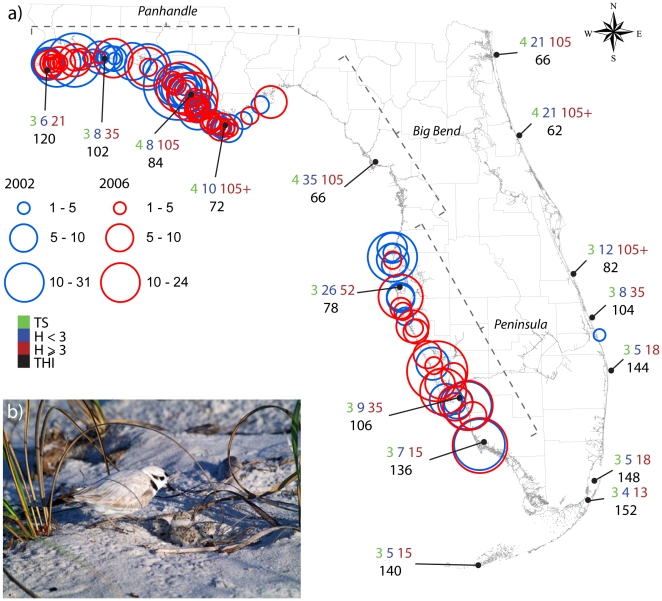
Wintering grounds and tropical cyclones frequencies. (a) Wintering distribution and abundance of Snowy Plover pairs in Florida in 2002 (blue circles) and 2006 (red circles) [51]. Green, blue, and red numbers are the average return periods for tropical storms, hurricanes, and severe hurricanes (category 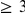). Black numbers represent the Tropical Hazard Index (THI) [34]. (b) SP male and three hatchlings close to the nest at the St. Joseph State Park, Apalachee Bay (credit to Raya Pruner). The apparent white plumage is an evidence of the strong life-history adaptation of SP to their niche [4], [5].

**Figure 2:**
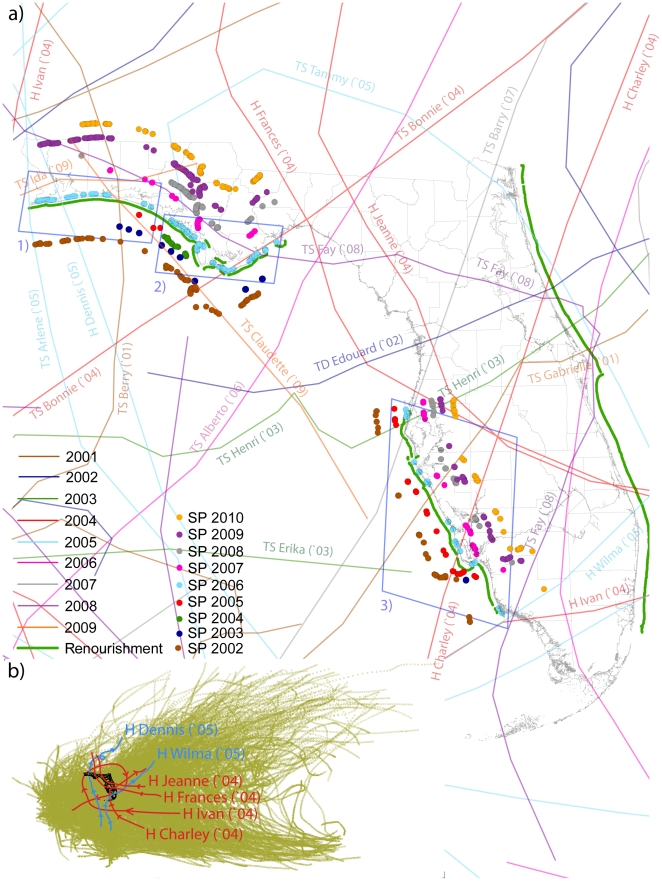
Nesting grounds and tropical cyclones tracks. (a) Nest ground locations of Snowy Plovers from 2002 to 2010 (dots) and tracks of the tropical cyclones (tropical depression (TD), tropical storm (TS), hurricane (H)) in the period 2001–2009 from [33]. An exploded view of the nest ground occurrences is proposed to avoid the overlaps of their locations along the coast for every year. The color match is between the nests and the cyclones in the previous year. (b) 1851–2009 hourly interpolated tracks/intensity [54] of the North Atlantic tropical cyclone database [33] that provides daily intensities. With typical clockwise trajectories, Atlantic basin cyclones form in the Atlantic ocean, make landfall in Central-North America, and return back into the ocean dissipating their energy [34], [48]. (1), (2), and (3) are the Northwest Florida, Apalachee Bay, and Southwest Florida regions in which the study is performed (see Table 1).

Figure 1 shows the winter distribution of SP in Florida and the return periods for tropical storms, hurricanes and major hurricanes [33]. Numbers in black are the Tropical Hazard Index (THI) derived from the sum of tropical cyclones from 1901 through 2005 [34]. The winter distribution of SP is similar to the breeding distribution (Figure 2 and Figure S2), which tends to coincide with the Florida coast most vulnerable to tropical cyclones (the Florida Panhandle and the southern Peninsula). These areas, highlighted in Figure 2, face the Gulf of Mexico and receive relatively more wind and water energy from tropical cyclones [35]. This natural beach re-nourishment provides new breeding and foraging grounds to SP (see Figure S1 for the pre- and post-hurricane habitat).

**Figure 3:**
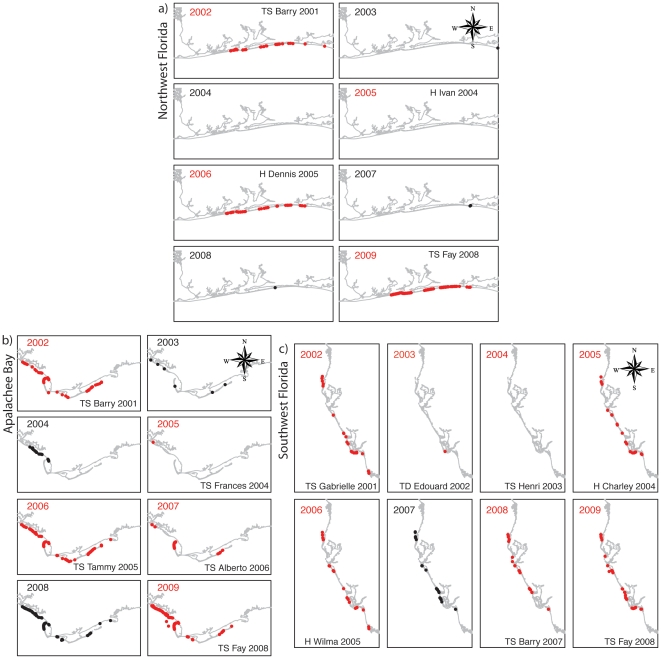
Spatial correspondence between SP nest distribution and cyclones in the previous season. Distribution of Snowy Plover nest sites by year for the north-west (NW) gulf coast (a), for the Apalachee Bay (b), and for the south-west (SW) Gulf coast (c) (1, 2, and 3 in Figure 2). A dot may represent more than a single nesting site. Left-corner text of the year following a tropical cyclone and the corresponding nests, are shown in red with the name of the strongest cyclone of the previous year given inside the panel. Text of year and the corresponding nests are shown in black if in the previous year no relevant tropical cyclone occurred. The classification of cyclones into tropical depressions (TD), tropical storms (TS), or hurricanes (H) is according their maximum wind speed within each of the three coastal regions considered.

Figures 3 shows the annual variation in nesting sites and their relationship to the occurrence of tropical cyclones for the three Florida regions considered along the Panhandle (NW coast, Apalachee Bay) and Peninsula (SW coast). The three regions are identified in Figure 2. The size of a region is dictated by a typical impact area for a large tropical cyclone. Tropical cyclones tracks that fall within a 50 km radius from the breeding range in each region are considered. The plots indicate that SP nesting sites are more common during the spring following a tropical cyclone affecting the region (red points).

To quantify this apparent relationship we used a Monte Carlo procedure to sample from the posterior distribution of the binomial probabilities that a region is a nesting ground conditional on whether or not the previous year experienced a tropical cyclone. Table 1 lists the number of nests by year and the tropical cyclone names from the previous year, if any. The median number of nests per year, per region is 57. Here we define a nesting ground as a region having at least 10 nesting sites. The regions are considered independent because they are geomorphologically and climatologically different [25], [34], [36], [37]. A mild “breeding independence” among the regions can be claimed because of the site-fidelity of the SP and the small dispersal range of the species [16], [20], [22]. We consider the region to have experienced a tropical cyclone if at least one named storm had winds of at least 17 m s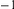 in the region.

**Table 1:** Snowy Plover nests and tropical cyclone occurrence.

Year	No. Nests	Tropical Cyclone Name (Intensity)
**Northwest Florida (** 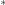 **)**		
2002	43	TS Barry (31 m s 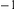 )
2003	2	
2004	0	
2005	0	H3 Ivan (57 m s 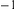 )
2006	52	TS Arlene (28 m s 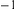 )/H3Dennis (57 m s 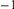 )
2007	3	
2008	0	
2009	422	TS Fay (21 m s 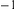 )
2010	48	TS Claudette (23 m s 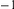 )/TS Ida (26 m s 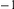 )
**Apalachee Bay (** 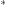 **)**		
2002	84	TS Barry (31 m s 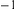 )
2003	7	
2004	57	
2005	1	TS Bonnie (23 m s 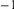 )/TS Frances (26 m s 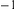 )
2006	177	TD Tammy (8 m s 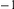 )
2007	42	TS Alberto (28 m s 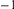 )
2008	393	
2009	622	TS Fay (23 m s 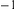 )
2010	193	TS Claudette (26 m s 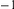 )
**Southwest Florida (** 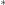 **)**		
2002	66	TS Gabrielle (31 m s 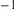 )
2003	1	TD Edouard (13 m s 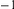 )
2004	0	TD Henry (15 m s 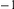 )/TS Erika (18 m s 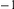 )
2005	94	H4 Charlie (64 m s 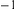 )/H1 Frances (41 m s 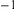 )/H2 Jeanne (49 m s 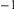 )
2006	69	H3 Wilma (57 m s 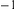 )
2007	97	
2008	79	TS Barry (23 m s 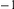 )
2009	196	TS Fay (28 m s 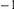 )
2010	66	

The number of Snowy Plover (SP) nests by breeding year (2002–2010) in three Gulf coastal regions of Florida. Names of the tropical cyclone in the year prior to the breeding year are given along with the cyclone's maximum intensity as it affected the region. In order of intensity quantified by the wind speed (SaffirÐSimpson scale): tropical depression (TD), tropical storm (TS, 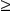
17 m s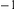), hurricane from category 1 to 4 (H1, H2, H3, H4, for intensity 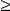
33, 43, 50, 59, and 70 m s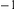, respectively). The nesting data are from the Florida Wildlife Commission [16], [17] and the tropical cyclone data are from the National Hurricane Center [33]. (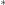) See Figure 2, highlighted regions (1), (2), and (3) for locations.

**Figure 4:**
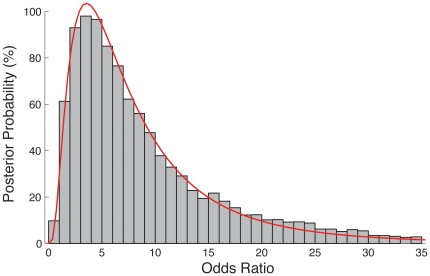
Posterior frequencies of the odds ratio. The odds ratio is the ratio of the odds of a nesting ground in the spring following a year with at least one tropical cyclone to the odds of a nesting ground in the spring following a year without a tropical cyclone. The frequencies are based on the relative frequency of the odds ratio being between the various class intervals along the horizontal axis from 10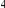 Monte Carlo samples. The median odds ratio is 7 and the mean is 11. The maximum likelihood estimate is a lognormal distribution (red curve) and the coefficient of determination is R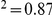.

Over the years 2002–2010 we have 13 cases of SP nesting grounds over a sample of 15 season-regions that were exposed to a tropical cyclone the year before, while we have 5 cases of nesting grounds over a sample of 12 season-regions that were not exposed to a tropical cyclone during the previous year. Assuming a uniform prior on the distributional parameters, we simulate posterior probabilities directly from the posterior distributions and compute the odds ratio as the fraction of the odds of a nesting ground in the spring following a year with at least one tropical cyclone, to the odds of a nesting ground in the spring following a year without a tropical cyclone (see Materials and Methods). Figure 4 is the posterior distribution of the odds ratio based on 10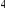 samples. A value above one indicates a relatively higher probability of a region being a nesting ground following a year with a tropical cyclone. The distribution is skewed to the right with a mode of between 3 and 4. The median value of the odds ratio indicates that it is 7 times more likely that a region will be a nesting ground following a year with a tropical cyclone impact. Given the data and the model, the probability that the odds ratio exceeds a value of two is 93%. A 90% confidence interval for the odds ratio is (1.8, 33), and the 95% confidence interval is (1.0, 35).

To further verify the relationship between TC and SP nest abundance in the year following the TC season, we used a logistic regression (see Materials and Methods). The model indicates that the occurrence of nesting grounds is more likely today than a decade ago and is significantly more likely in the spring that follows a tropical cyclone after controlling for the temporal trend (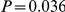). Expressed in terms of the odds ratio, the model indicates it is 8.5 times more likely for a region to be a nesting ground for SP in years following a tropical cyclone compared to years without a cyclone.

## Discussion

Land-cover change due to sea-level rise, as predicted by climate change models, could be one of the most prominent hazards threatening the fate of SP in Florida [25]. Nonetheless, direct human interventions such as disturbance, and nourishment projects not intended to restore species habitat, as well as anthropogenic catastrophes like oil spills [38], pose a serious risk to SP survival and for the whole coastal ecosystem in general. Assessing macroecological relationships between species and climatic features is very important to understand the response of biodiversity under future climate change scenarios. The response of the “Elthonian” niche [39], that is defined in terms of interactive environmental variables and biological features at broad scales, is clearly a function of the response of the habitat to climatological changes.

We have detected the spatio-temporal relationships between tropical cyclones events and SP nesting abundance and range in the years following a TC season. Figure S2 shows how SP are mainly located on state and federal lands in Florida that act as hotspots of biodiversity. While we state a causal interannual relationship between TC and SP abundance in the year following a cyclone event, we do not diminish the importance of the land-cover in determining favorable habitat, or the influence of anthropic disturbances on the distribution of nesting sites for SP. State and federally protected lands clearly provide a more protected habitat with less human disturbance for shoreline-dependent birds than public areas. Nonetheless SP also nest in locations with dense human populations populated (along the Peninsula) and in tourist locations (Panhandle and Peninsula). Approximatively 58% of the SP population inhabits the protected state and federal lands along the Florida coast. However the creation of new conservation areas does not imply directly that new nesting habitat will be created. Many state, and federal shorelines, including coastal military installations (e.g. along the Atlantic coast) are not populated by SP. This is largely explainable by the lack of favorable habitat along those shores [25].

The logistic regression overestimated the probability of finding a nesting ground in the season that follows the tropical cyclone season in the previous year. The Bayesian approach provided instead a rational and coherent foundation for using all the available information, while explicitly accounting for differences in uncertainty and sampling effort of data [40]. The Bayesian approach is not new in modeling tropical cyclones [41] and has been adopted in our study, for the first time, to investigate the interannual feedback between TC and SP abundance and range. The strong positive bioclimatic feedback lends strong evidence that TC effects can be largely beneficial for some species, which refutes some generalist catastrophic assessments about TC on ecosystems [42], [43]. Figure S1 shows that within the Santa Rosa barrier island (box (1) in Figure S2) [37], [44], the hurricanes of 2004 and 2005 caused the island to change from a discontinuous foredune backed by hummocky backbarrier dunes and maritime forest (at the bayside cuspate headlands) to washover terraces at the headlands and washover corridors between headlands [44]. [44] found that on average the beachfront recovery and the backshore accretion (estuary and hind-dune areas) in the Santa Rosa unit, occurred at the widest sections (“cuspate headland” section in Figure S1) of the island where the pre-storm profile sand volume had been relatively large and overwash penetration was at a minimum. This dynamic seems to be in common for all the barrier islands during cyclones events (tropical storms, hurricanes, and major hurricanes). The higher percentage of tidal flat areas with an increased open-view of the habitat [45], presence of diffuse debris, and large amount of organic material on the beach increase the favorability of these sites as nesting areas for SP [25].

Land-cover, climate, and human disturbance are the main drivers that simultaneously shape SP nesting distribution. The relationship of SP abundance (proportional to the number of nesting grounds) and TC is one of the mechanisms that appears evident in determining the range of the SP population. [25] have identified the Grinnellian niche [39] for the SP in Florida: white alkaline sandy estuarine/ocean beaches. This constitutes one of the linkages between the geomorphology of the habitat and the ecological dynamics of SP. The shore, beach and dune changes caused by TC is positive for SP and may also be favorable for other shoreline-dependent birds like Piping Plovers and Red Knots [5], [6], [26] which use similar habitats of the SP [46], [47] during the winter and for their migration to central and south America wintering sites. The investigated feedback can be possibly valid in other areas subjected to cyclones, for shorebirds that benefit from the creation of new breeding and foraging areas. Further studies should investigate in detail multiple-species patterns relative to tropical cyclones, also at larger continental scales.

While untangling the biogeoclimatology of SP in Florida, we found the almost paradoxical result that SP benefits from tropical cyclones through habitat creation and maintenance. In general, cyclones are hazardous for human populations and infrastructures, however they shape ecosystems [30] and assist in maintaining shorebird biodiversity patterns, including the abundance and range of SP. Today 20% of the strongest cyclones have winds exceeding 49 m s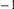 on average globally. With a 1 rise in sea-surface temperature, 20% of the strongest cyclones will exceed 51 m s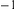 according to [48]. Thus the 80th percentile increases from 49 to 51 m s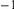. Today, on average, 17 cyclones/year exceed 49 m s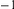 and 13 exceed 51 m s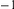. If 51 m s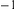 is the new 80th percentile (after a 1°C warming) then, without a change in the overall number of cyclones, the number of cyclones that exceed 51 m s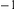 increases from 13 to 17. Since future climate change scenarios depict fewer but fiercer tropical cyclone events [48]–[50], the widespread geomorphological structuring of the beach habitat could foreseeably be reduced. This can constitute a negative feedback on the sensitive SP population in Florida and on other shorebirds species with similar habitat preferences, while amplifying the dangers to local human populations.

## Materials and Methods

### Ecobiogeographical data

The collection of SP data at the landscape and nest-site scale was performed through the years by Florida Wildlife Commission (FWC) personnel, volunteers, landowners, military personnel, and state/national park personnel. The data sources reported in Table 1 are [16], [17], [51]. The sampling effort in 2003, 2004, and 2005, was not as extensive as for the other years. In 2002 the SP sampling was initiated by the USGS-International Piping Plover Census (IPPC) [51], and in 2004–2005 the sampling was limited by the devastating cyclone season [52]. The scenopoetic and bionomic variables collected in the field from these campaigns are numerous (e.g., the distance of nests from the high tide line, from the front dune line, to the nearest vegetation patch, and to the nearest structural debris [25], [46]). This study utilizes solely the GPS coordinates of the nests measured in the field.

The effects of tropical cyclones on coastal areas are strongest in a range of (30, 60) km from their main track, that describes on average the movement of the “cyclone's eye” [34], [53]. A tropical cyclone is a storm system characterized by a large low-pressure center and numerous thunderstorms that produce strong winds and heavy rain. Atlantic basin cyclones (Figure 2, b) form in the Atlantic ocean, often making landfall in the USA. As they do so, speeds typically increase until landfall, then decrease across land as they move in a clockwise trajectory back into the Atlantic [34], [48]. Cyclones that have a wind speed of at least 17 m s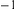 (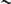
34 knots) are included in the analysis. A mean distance of 50 km was considered from the nests detected for each subpopulation for defining the three regions in our study (Apalachee Bay, NW and SW Florida). We use either the hourly interpolated points (Figure 2, b) or the daily tracks polylines (Figure 1 and Figure 2, a).

The Apalachee Bay, NW and SW Florida SP population represents 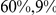, and 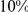, respectively, of the whole Florida SP population. The delineation of the study regions was completed assuming the geomorphological and climatological independence of the three areas. The areas are characterized by different elevation, erosion and accretion rates determined by their geology and the ocean currents [25], [37], [46]. The NW region is quite stable, but the Apalachee Bay and the SW Florida regions are very dynamic coastal areas [25], [36], [37]. As for climatology, the three regions are characterized by different return periods of cyclone phenomena and temperature [34] (Figure 1). The breeding biology of SP is not only dynamic but also complex. SP, that shows a high degree of philopatry [16], [20], [22], can have two to three mating bouts in a season [16], [17]. However, the “breeding independence” of the regions is not a necessary condition of the model. In fact SP are likely attracted by the new suitable nesting grounds created by tropical cyclones. The model is a posteriori inference between nesting grounds abundance and the occurrence of cyclone events in the previous year of the breeding season considered.

### Bayesian Inference

A Bayesian inference with Monte Carlo sampling has been used to detect the causality between tropical cyclones and Snowy Plovers nesting grounds. Let 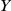 be a random variable having a value of one if the region is a nesting ground and zero otherwise and 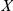 be a random variable having a value of one if the region was affected by a tropical cyclone in the previous year and zero otherwise. Then the odds ratio (OR) of a region being a nesting ground given at least one tropical cyclone the year before relative to the region being a nesting ground following a year without a tropical cyclone is given by
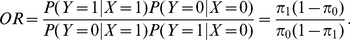
(1)


The likelihood function given by the product of binomial distributions 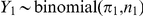 and 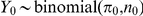. Assuming beta priors for the probabilities 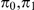 with parameters 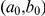 and 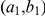, our posteriors are given by 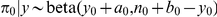 and 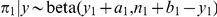.

We have 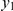 = 13 cases of SP nesting grounds over a sample of 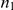 = 15 breeding seasons that were exposed to a tropical cyclone with winds exceeding 20 m s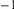 the year before, while we have 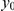 = 5 cases of nesting grounds over a sample of 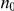 = 12 breeding seasons that were not exposed to a previous year tropical cyclone. Assuming a uniform prior (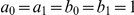), we simulate 10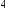 posterior probabilities directly from the posterior distributions 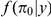 and 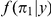 and compute the OR as given in Eq. 1. Statistics on the OR follow directly from the posterior samples.

### Logistic Regression

The dependent variable is whether or not the region can be considered a nesting ground. A nesting ground is defined as having at least 10 nesting sites. The logistic regression models this dichotomous variable as a function of year and the occurrence or not of a tropical cyclone, in the cyclone season prior to breeding.

Logistic regression is a statistical model used here to predict the annual occurrence of SP breeding. There are three potential breeding regions and 10 years (from 2002 to 2010) for each region for a total of 30 cases. The breeding year 1989 was not considered due to the high uncertainty in the collection of the data [16], [17]. Breeding year is included as an independent variable. A maximum likelihood technique is used to obtain the model coefficients. The model can be expressed as:
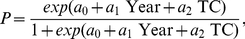
(2)where 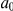 is the intercept term, 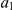 is the coefficient indicating a temporal trend, 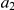 is the coefficient indicating the relationship of breeding to the occurrence of a tropical cyclone, and 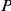 is the probability of a group of SP nests (breeding region) in the season.

## Supporting Information

Figure S1 **Pre-post cyclone pictures of a typical barrier island.** Aerial pictures of the Santa Rosa Island unit (box (1) in Figure S2) for the pre- and post-hurricanes Ivan, Dennis, and Katrina event [44]. The left pictures are for a “cuspate headland” section that is the widest section of the island, and the right pictures are for the narrower “between headland” section. In the pictures the back of the barrier island is indicated. (Credit to C. Houser for the aerial pictures).
(TIF)

Figure S2 **Correspondence between federal lands and SP nests.** and (a) Exploded view of the Snowy Plover nesting sites from 2002 to 2010 (red dots) [16], [17], and delineation of protected military sites, state, and national parks. (1) unit of the Santa Rosa Island. (b) zoom for the breeding range along the Panhandle. (c) zoom for the breeding range along the Peninsula.
(TIF)
