# Supplementary figures and images for: Do Tropical Cyclones Shape Shorebird Habitat Patterns? Biogeoclimatology of Snowy Plovers in Florida
Source: PLoS One. 2011 Jan 12;6(1):e15683. doi: 10.1371/journal.pone.0015683 (PMC3020223; doi:10.1371/journal.pone.0015683)

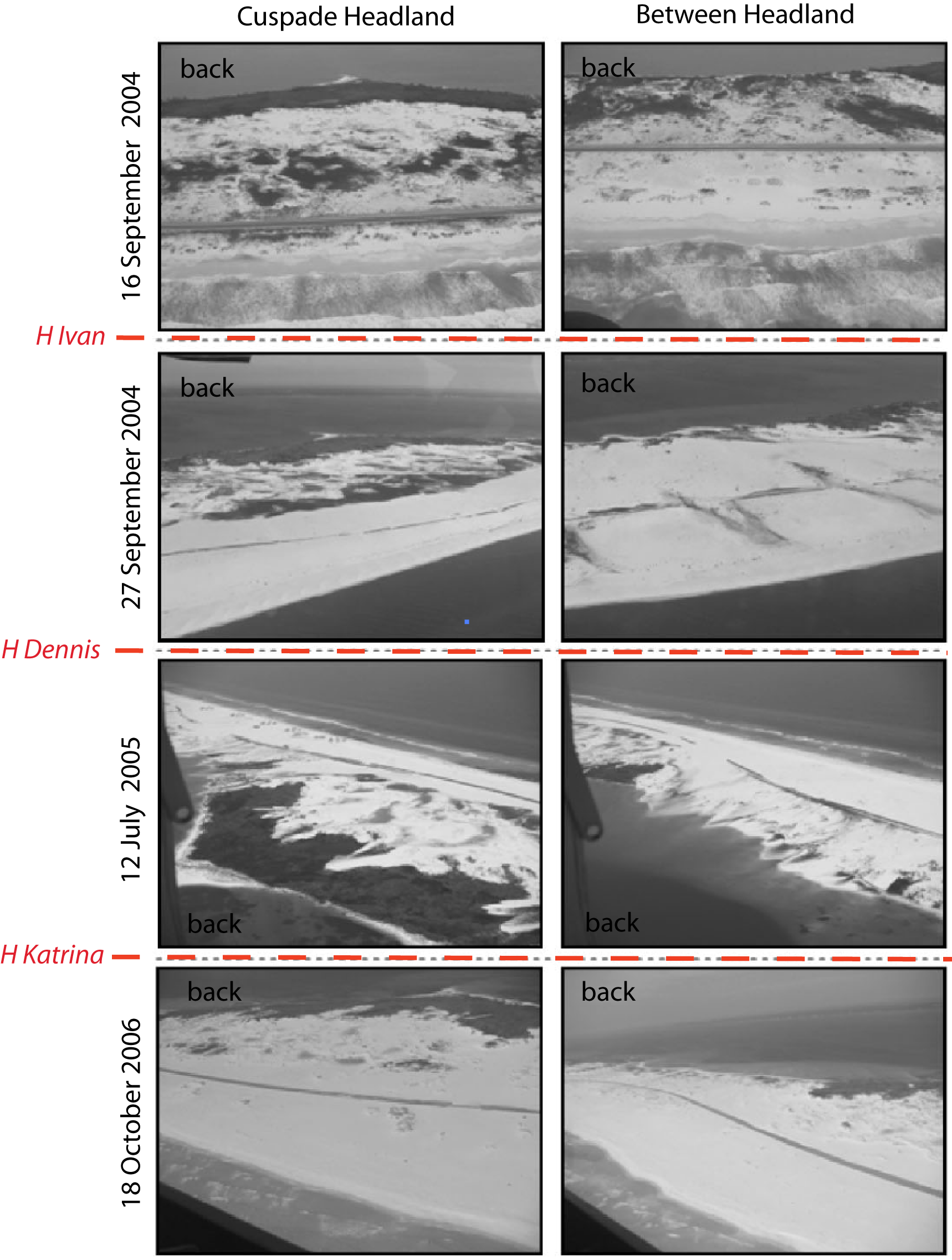

Supplement: Figure S1 — Pre-post cyclone pictures of a typical barrier island. Aerial pictures of the Santa Rosa Island unit (box (1) in Figure S2) for the pre- and post-hurricanes Ivan, Dennis, and Katrina event [44]. The left pictures are for a “cuspate headland” section that is the widest section of the island, and the right pictures are for the narrower “between headland” section. In the pictures the back of the barrier island is indicated. (Credit to C. Houser for the aerial pictures). (TIF) [file pone.0015683.s001.tif]

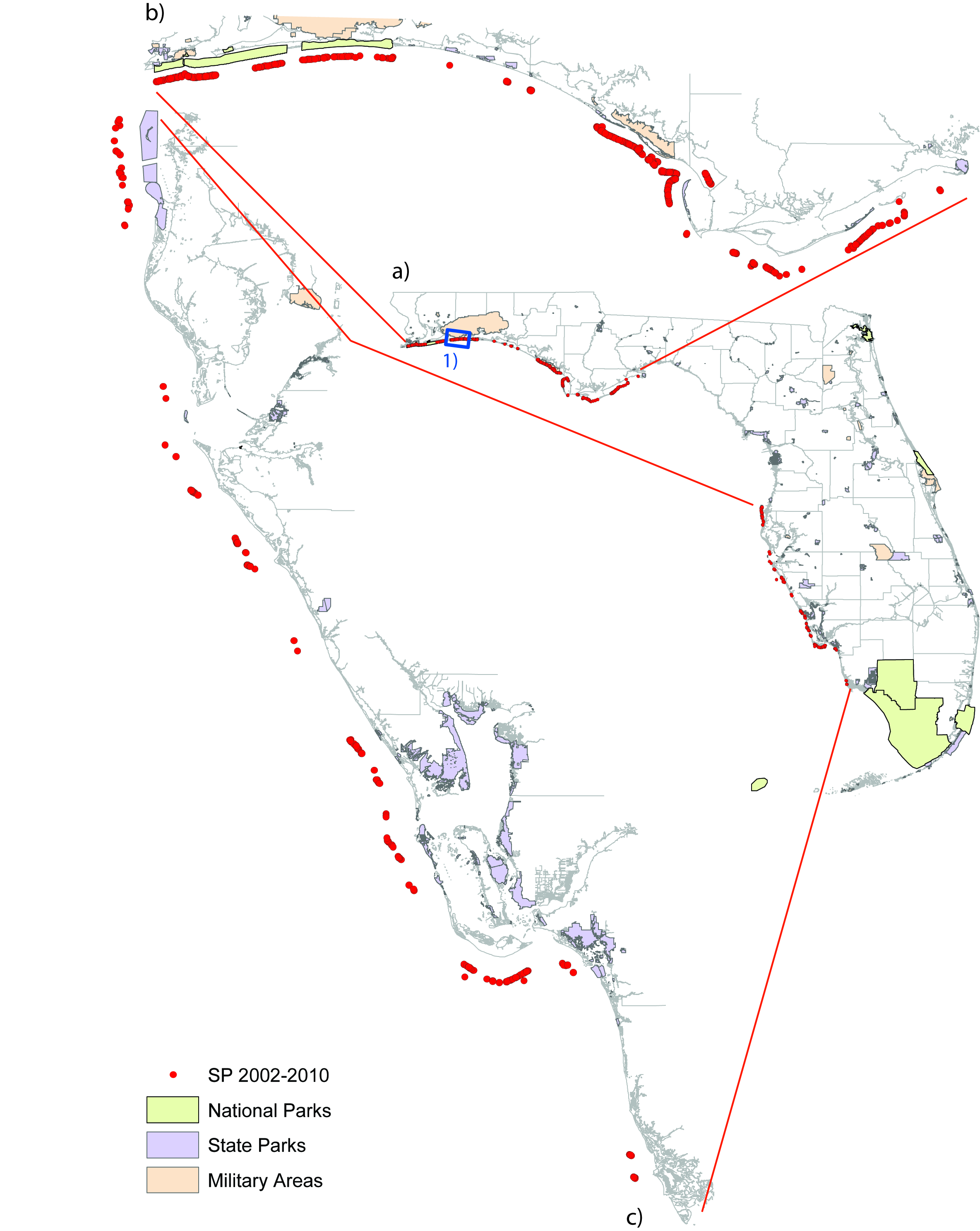

Supplement: Figure S2 — Correspondence between federal lands and SP nests. and (a) Exploded view of the Snowy Plover nesting sites from 2002 to 2010 (red dots) [16], [17], and delineation of protected military sites, state, and national parks. (1) unit of the Santa Rosa Island. (b) zoom for the breeding range along the Panhandle. (c) zoom for the breeding range along the Peninsula. (TIF) [file pone.0015683.s002.tif]
